# The Role of Haptoglobin Genotypes in Chagas Disease

**DOI:** 10.1155/2014/793646

**Published:** 2014-07-24

**Authors:** Ninomar Mundaray Fernández, Mercedes Fernández-Mestre

**Affiliations:** Laboratorio de Fisiopatología, Centro de Medicina Experimental “Miguel Layrisse”, Instituto Venezolano de Investigaciones Científicas, Kilómetro 11 Carretera Panamericana, Apartado 21827, Caracas 1020A, Venezuela

## Abstract

Although the number of people infected with *T. cruzi* is on the rise, host genetic and immune components that are crucial in the development of the Chagas disease have been discovered. We investigated the frequency of polymorphisms in the gene encoding haptoglobin of patients with chronic Chagas disease. The results suggest that while the *HP1-1* genotype may confer protection against infection and the development of chronic Chagas disease due to the rapid metabolism of the Hp1-1-Hb complex and its anti-inflammatory activity, the presence of *HP2-2* genotype may increase susceptibility towards a chronic condition of the disease due to a slow metabolism of the Hp2-2-Hb complex, lower antioxidant activity, and increased inflammatory reactivity, which lead to cell damage and a deterioration of the cardiac function. Finally, correlations between *HP* genotypes in different age groups and cardiac manifestations suggest that *HP* polymorphism could influence the prognosis of this infectious disease. This study shows some of the relevant aspects of the haptoglobin gene polymorphism and its implications in the *T. cruzi* infection.

## 1. Introduction

American trypanosomiasis is recognized by the World Health Organization (WHO) as one of the 13 major neglected diseases in the tropical world constituting a serious social and economic impact in several countries, especially in Latin America. In the world today, this disease accounts for 16–18 million cases, 21,000 annual deaths, 2-3 million cases with chronic complications, approximately 300,000 new infections each year, and over 80 million people at risk of being infected [1]. Several studies suggest that haptoglobin polymorphisms may play an important role in the immune response against various infectious diseases because of their oxidizing action and immunomodulatory properties in other pathologies. However, depending on the population under study, the associations between* HP* genotypes and diseases can be controversial due to the large number of variables involved such as the ethnic composition and homogeneity of a given population, the number of individuals studied, differing genotyping methodologies, and the pathophysiologic complexity of the related disease.

Haptoglobin (Hp) is an acute-phase protein synthesized mainly by the liver during inflammatory processes whose main function is to remove the free hemoglobin (Hb) by forming Hp-Hb complexes that are eliminated primarily by monocytes/macrophages. Additionally, haptoglobin also possesses anti-inflammatory and antioxidant properties [2]. The haptoglobin polymorphism is composed of two* HP1* and* HP2* codominant alleles resulting in three major* HP* genotypes* HP1-1*,* HP2-2*, and* HP2-1* corresponding to proteins of different structural and functional properties [3]. The Hp 1-1 phenotype has been associated with high plasma concentrations [4] and an increased antioxidant capacity [5], affinity for hemoglobin [4], and clearance of Hp-Hb (CD163-mediated) [6] regarding Hp phenotype 2-2. The Hp 1-1 phenotype also induces greater amounts of anti-inflammatory interleukins than the Hp 2-2 phenotype [7].

Only two studies have so far addressed the possible association between* HP* polymorphism and the pathogenesis of Chagas disease. In the first study, Calderoni et al. suggested that Hp phenotypes could be associated with different clinical forms of American trypanosomiasis. In comparing the frequency of Hp phenotypes between patients and healthy individuals they found that the Hp2-2 phenotype was more frequent in patients with any of the clinical forms of the disease (*P* = 0.0001 for each form), suggesting that the Hp 2-2 phenotype in individuals exposed to* T. cruzi* could favor the development of disease pathology [8]. In the second study, Jorge et al. investigated the possible association between* HP* genotypes and the severity of cardiac complications in patients with Chagas disease. Their study found that while the* HP2* allele appears to confer increased risk for developing serious complications of heart disease, those patients with genotype* HP1-1* were less likely to develop the heart disease, implying that* HP* polymorphism may influence the clinical course of Chagas disease [9].

In Venezuela, the number of cases of* T. cruzi* is steadily growing. Recent studies have shown that genetic and immunological components of the host are crucial elements in the development of the cardiac form of the disease and that genetic factors may serve as prognostic tools in the development and pathologic severity in patients with Chagas disease. Therefore, because of the role of haptoglobin in the immune response and previous studies pointing to an association of* HP* genotypes with Chagas disease progression, we investigated the association of haptoglobin gene polymorphisms in Venezuelan patients with chronic Chagas disease.

## 2. Material and Methods

### 2.1. Subjects

Our study included two hundred and sixteen (216) unrelated and ethnically mixed Venezuelan individuals from the northern region of country whom we classified into 2 groups. Chagasic patients (*n* = 96): individuals seropositive for* T. cruzi* were followed by the “Centro de Investigaciones José Francisco Torrealba” in San Juan de los Morros, Guárico State, from 1995 to 1998. The diagnosis of infection was performed by the complement fixation test, molecular methods (PCR), and, in some cases, indirect haemagglutination and xenodiagnosis. The patients with Chagas disease were classified according to clinical and electrocardiographic (ECG) characteristics [10] as asymptomatic patients without cardiac symptoms (Group A: *n* = 30, 57% men, 43% women; age range: 33–54 years), patients with arrhythmia-related symptoms (mild heart disease or Group B: *n* = 30, 57% men, 43% women; age range: 54–65 years), and patients with overt congestive heart failure (severe heart disease or Group C: *n* = 36, 72% men, 28% women; age range: 65–86 years). Notably, although the proportion of female (28%) to male (72%) is minor in the severe disease group, when compared to the other two groups of patients (57% versus 43%, resp.) this difference is not attributable to longer survival or a predominance of this disease to a specific gender.

Controls (*n* = 120): unrelated healthy subjects of similar ethnic background, seronegative for* T. cruzi* (molecular and serological methods) and other infectious diseases (HIV, Hepatitis C, Syphilis), were tested as controls (68% men, 32% women; age range: 17–58 years).

Those individuals who agreed to participate in the study were asked to sign an informed consent form which, in turn, was submitted to and subsequently approved by the IVIC Bioethics Committee prior to the onset of the investigation.

### 2.2. Haptoglobin Genotype Analysis

Genomic DNA was extracted from blood samples following the procedure described by Bunce [11] and the haptoglobin genotyping was performed by allele-specific polymerase chain reaction according to Koch et al. [12]. PCR was performed on a 20 *μ*L reaction mixture containing 30 ng/*μ*L DNA, 3.3 mM MgCl2, 0.4 mM Tris HCl, 2.5 mM KCl, 0.2 mM dNTP mix, 0.2 *μ*M of each primer (*Hp1* allele: A and B primer;* Hp2* allele: C and D), 0.1 *μ*M of each *β*-globin primer, and 1.25 units of Platinum Taq DNA polymerase (Invitrogen Life Technologies, Sao Paulo, Brazil). After initial denaturation at 95°C for 2 min, the two-step thermocycling procedure consisted of denaturation at 95°C for 1 min and annealing and extension at 69°C for 2 min (in the presence of primers A and B) or 1 min (in the presence of primers C and D only), repeated for 35 cycles and followed by a final extension at 72°C for 7 min. The thermocycler used was GeneAmp PCR systems 9700 (Applied Biosystems). For genotype assignments, the PCR products were separated in 1.5% agarose gels.

### 2.3. Statistical Analysis

Allele and genotype frequencies were determined by direct counting. The Hardy-Weinberg equilibrium was calculated through a chi-squared test. The statistical significance of allele frequency differences between patients and controls was estimated by Fisher's exact test using 2 × 2 contingency tables. Relative risk with corresponding 95% confidence intervals (95% CI) was calculated as odds ratios (OR) according to Woolf's formula [13]. Multiple logistic regressions were performed to determine whether age and Hp genotypes were associated with the prognosis of the disease.

## 3. Results

### 3.1. Frequency of the* HP* Polymorphism in Patients with Chronic Chagas Disease and Healthy Individuals

The analysis allowed us to establish the allelic and genotypic frequencies of the* HP* polymorphisms. The findings suggest the existence of a Hardy-Weinberg equilibrium for genotype distribution in both patients (*X* ^2^ = 1.19; *P* > 0.05) and healthy individuals (*X* ^2^ = 3.66; *P* > 0.05).  Table 1 shows the frequencies of* HP* genotypes in controls and chagasic patients. In comparing the frequencies of the genotypes between the groups studied, a significantly increased frequency of* HP1-1* genotype in healthy individuals versus chagasic patients was observed (18% versus 4%, resp., OR = 0.20, 95% CI: 0.068–0.620, *P* = 0.002, *P*_*c*_ = 0.006). No difference in the distribution of the allele frequencies was found among controls and chagasic patients.

### 3.2. Frequency of the* HP* Polymorphism in Healthy Individuals and Chagasic Patients with Cardiac Disorders (Mild and Severe Heart Disease)

Significant differences were observed in the comparison of genotypic and allelic frequencies between chagasic patients with cardiac disorders (Groups B and C) and healthy individuals (Table 2). The* HP1-1* genotype was higher in healthy individuals (18% in healthy versus 5% in symptomatic, OR: 0.22, 95% CI: 0.064–0.7837, *P* = 0,01, *P*_*c*_ = 0.03) and the* HP2-2* genotype was significantly higher in symptomatic chagasic patients (Groups B + C) (71% in symptomatic versus 44% in healthy; OR: 3.13, 95% CI: 1.6436–5.9493, *P* = 0.0003, *P*_*c*_ = 0.0009). In addition, we observed that there was a significantly higher frequency of the* HP1* allele in healthy individuals (37% in healthy versus 17% in symptomatic, OR: 0.35, 95% CI: 0.2038–0.5855, *P* = 0.00004, *P*_*c*_ = 0.00008) and a significantly higher frequency of* HP2* allele in chagasic patients with cardiac abnormalities (83% in symptomatic versus 63% in healthy, OR: 2.89, 95% CI: 1.7078–4.9064, *P* = 0.00004, *P*_*c*_ = 0.00008).

### 3.3. Frequency of the* HP* Polymorphism in Symptomatic and Asymptomatic Chagasic Patients

The distribution of* HP* genotype frequencies in the different groups of patients with Chagas showed a significantly higher frequency of the* HP2-2* genotype in symptomatic versus asymptomatic patients (71% in symptomatic versus 17% in asymptomatic, OR: 12.37, 95% CI: 4.1251–37.0845, *P* = 0.000001, *P*_*c*_ = 0.000003). Furthermore, there was a significantly higher frequency of the* HP2* allele in symptomatic patients (83% in symptomatic versus 57% in asymptomatic, OR: 3.82, 95% CI: 1.9260–7.5904, *P* = 0.00008, *P*_*c*_ = 0.00016) and a significant increase of the* HP1* allele in asymptomatic patients (43% in asymptomatic versus 17% in symptomatic, OR: 0.26, 95% CI: 0.1317–0.5192, *P* = 0.00008, *P*_*c*_ = 0.00016) was found (Table 3).

### 3.4. Correlation between Age,* HP* Genotype, and Clinical Manifestations in Patients with Chronic Chagas Disease

**Figure 1:**
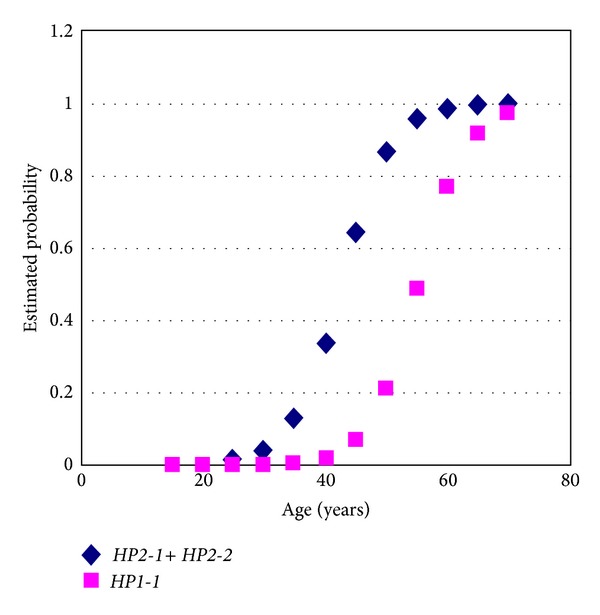
Probability estimates for developing chronic Chagas disease in patients with genotypes* HP1-1* and* HP2-1* +* HP2-2* by age. Individuals with* HP1-1* genotype are less likely to develop the disease at ages below 50 while individuals with at least one* HP2* allele are more likely to present any clinical form of the disease at younger ages.

By correlating the susceptibility to develop chronic disease in any of its clinical forms, with age and* HP* genotype, we observed that individuals with genotype* HP1-1* are less likely to develop the disease under the age of 50 while individuals with one or two* HP2* alleles are more likely to present any clinical form of the disease at younger ages. However, in older individuals (about 70 years) the* HP* genotype does not affect the development of the clinical forms of the disease (Figure 1).

## 4. Discussion

The haptoglobin genotypes have phenotypic, biochemical, and biophysical differences affecting their antioxidant and immunomodulating functional properties. Results from clinical studies have shown that* HP* gene polymorphisms can exert various effects on the course of bacterial, viral, and parasitic infections, as well as noninfectious diseases, affecting the severity and progression of the pathology [14]. In chronic Chagas disease, one of the most important objectives of the clinical study was to determine why some infected individuals develop severe manifestations while others remain asymptomatic. One possible theory is that the regulation that controls the immune response is virtually absent in patients with chronic Chagas heart disease [15]. In the present study we observed that the* HP2-2* genotype and the* HP2* allele predominated in both patients and healthy individuals, but comparisons of the frequencies of* HP* genotypes between infected and healthy individuals showed significant differences. The* HP1-1* genotype had a significantly increased frequency in healthy individuals, suggesting that this genotype confers protection against infection by* T. cruzi*. Individuals with the* HP1-1* genotype produce higher concentrations of the Hp protein, which has increased binding affinity for the hemoglobin and may thus prevent iron intake required by trypanosomes for replication.* In vitro* cultures demonstrated that* Trypanosoma cruzi* and* Trypanosoma brucei* growth require the adding of heme compounds to the medium in the form of hemoglobin, hematin, or heme. The genome sequencing of* T. cruzi* and* T. brucei* (TriTrypDB, http://tritrypdb.org/tritrypdb/) confirmed the absence of genes for enzymes involved in the biosynthesis of heme in the genomes. Therefore, they must scavenge this molecule from their hosts [16]. The rapid metabolism of the Hp1-1-Hb complexes could be preventing the intake of heme compounds and, with it, the iron required for optimal growth of* Trypanosoma cruzi* in the host cells. Loo and Lalonde [17] suggested that intracellular iron depletion in the host could protect against infection by* T. cruzi* whereas anything that results in the transfer of transfer iron to intracellular sites would promote the replication of* T. cruzi* and could thus increase the pathogenicity of the parasite.

On the other hand, the* HP2-2* genotype had a significantly higher frequency in chagasic patients with mild and severe cardiac symptoms (Groups B and C, resp.), suggesting that this genotype confers susceptibility to the cardiac disease characteristic of Chagas disease. This opinion was confirmed in comparisons between symptomatic and asymptomatic Chagas infected patients.

To explain this susceptibility promoted by the* HP2-2* genotype, we considered two important aspects: (1) trypanosomatids requiring iron for vital processes including DNA replication and mitochondrial respiration; the heme biosynthetic pathway is completely absent in* T. cruzi*, so the iron must be obtained from the host [16]; (2) the structural and functional characteristics of the molecule Hp 2-2. Individuals with* HP2-2* genotype produce lower protein concentrations (0.38 to 1.50 g/L), the molecule Hp2-2 [4]. This molecule is larger with a less effective uptake capacity of Hb. Therefore the Hp2-2-Hb complexes are metabolized more slowly and the free hemoglobin remains for longer in circulation. Furthermore, the multimeric complex Hp2-2-Hb is captured with a higher affinity for CD163 receptors on macrophages, which, to process the complex, increase the induction of ferritin synthesis and thus the availability of iron in the host [18, 19]. Finally, an individual with phenotype Hp2-2 develops lower protective activity against free radicals which promotes an inflammatory response (Th1). Therefore, the functional properties of the* HP2* allele, such as a lower concentration of haptoglobin in circulation, a decreased antioxidant activity, a slow metabolism of Hp2-2-Hb complexes (favoring the availability of iron) and promote a cytokine profile Th1 establishes a pro-oxidant microenvironment following the infectious process. This sustained production of reactive oxygen species (ROS) initially reduces the parasite load in symptomatic patients. However, with the passage of time, diseased patients generate an exacerbated inflammatory response (Th1) that cannot be controlled, thus leading to cell damage and a subsequent deterioration of heart function. During the course of* T. cruzi* infection and the development of the disease, the ROS can be produced as a consequence of tissue destruction caused by the toxic secretions of the parasite, immune cytotoxic reactions, and secondary damage to the mitochondria [20]. Therefore, the low antioxidant capacity in individuals with a Hp2-2 phenotype could favor the pathophysiological damage of the heart tissue of chagasic patients by promoting increased formation of ROS, the establishment of oxidative stress, inducing a Th1 response characterized by the inflammatory cytokine expression.

In consequence the antioxidant therapy might benefit patients with* HP2-2* genotype and chronic Chagas disease who are treated with adrenergic blockers approved for the treatment of heart failure and left ventricular dysfunction. Thus the use of antioxidants in chagasic patients with* HP2-2* genotype potentially could alter the clinical course of their disease.

Our results are consistent with two studies conducted in Brazil, the first of which determined the frequency of the haptoglobin phenotypes in chagasic patients and healthy individuals, presenting the Hp2-2 phenotype (with a lower antioxidant capacity and increased inflammatory reactivity) with an increased frequency in patients compared to healthy individuals [8]. The second study investigated the possible association between haptoglobin genotypes and the severity of the Chagas disease [9]. Although no significant differences were observed in the distribution of genotype frequencies between patients and controls, multiple logistic regressions showed correlations between* HP* genotypes in different age groups and cardiac manifestations (indeterminate, mild, and severe forms of trypanosomiasis).

The correlations were as follows.

1. Young patients with* HP1-1* genotypes were asymptomatic and the probability of developing mild heart condition with increasing age was greater than of developing severe heart disease. This coincides with the correlation found in our study where we observed that individuals with the genotype* HP1-1* were less likely to develop the disease before the age of 50.
2. Patients with the* HP2-2* genotype have a higher chance of developing heart manifestations earlier in their lives than patients with other genotypes. This is also very similar to the correlation found in our study where we observed that individuals with at least one allele* HP2* (*HP2-2*,* HP1-2*) were more likely to have any clinical form of the disease at younger ages.
3. Patients with genotype* HP2-1* have a higher chance of developing cardiac manifestations at younger age than* HP1-1*.
4. However, in our analysis of correlations between* HP* genotypes in different age groups and cardiac manifestations, we observed that among older individuals (about 70 years) the* HP* genotype does not affect the development of the clinical pathology.

In this study we have highlighted some of the more important aspects of the haptoglobin gene polymorphism and its implications in the* T. cruzi* infection.

## 5. Conclusions

The results obtained in our study suggest the following.

1. The* HP1-1* genotype may confer protection against the infection caused by the parasite* Trypanosoma cruzi* and the development of chronic Chagas disease due to its anti-inflammatory activity and the rapid metabolism of Hp1-1-Hb complexes.
2. The* HP2-2* genotype may confer susceptibility to the development of chronic Chagas disease due to a slow metabolism of Hp 2-2-Hb complexes, a lower antioxidant activity, and increased inflammatory reactivity, which would lead to cell damage and impaired cardiac function.
3. Correlations between* HP* genotypes in different age groups and cardiac manifestations suggest that* HP* polymorphism could influence the prognosis of this infectious disease.

## Figures and Tables

**Table 1:** *HP* genotype frequencies in healthy individuals and in chronic Chagas disease patients.

	Chagasic patients n = 96	Healthy individuals n = 120	OR	*P*_*c*_
Genotypes				
*HP 1-1 *	4 (4)	18 (21)	0.2	0,006∗
*HP 2-1 *	42 (40)	38 (46)	1.15	ns
*HP 2-2 *	54 (52)	44 (53)	1.49	ns
Alleles				
*HP1 *	25 (48)	37 (88)	0.71	ns
*HP2 *	75 (144)	63 (152)	1.41	ns

*Note*. The genotype and alleles frequencies are expressed in percentages, followed by the number of individuals or chromosomes in parentheses. OR: odds ratio, *P*_*c*_: corrected *P* values, ns: not significant, ∗significant.

**Table 2:** *HP* genotype frequencies in healthy individuals and patients with chagasic cardiac abnormalities (symptomatic).

	Symptomatic (B + C Groups) *n* = 66	Healthy individuals *n* = 120	OR	*P*_*c*_
Genotypes				
*HP 1-1 *	5 (3)	18 (21)	0.22	0,03∗
*HP 2-1 *	24 (16)	38 (46)	0.69	ns
*HP 2-2 *	71 (47)	44 (53)	3.13	0,001∗
Alleles				
*HP1 *	17 (22)	37 (88)	0.35	0,00008∗
*HP2 *	83 (110)	63 (152)	2.89	0,00008∗

*Note*. The genotype and alleles frequencies are expressed in percentages, followed by the number of individuals or chromosomes in parentheses. OR: odds ratio, *P*_*c*_: corrected *P* values, ns: not significant, ∗significant.

**Table 3:** *HP* genotypic frequencies in symptomatic and asymptomatic patients with Chagas.

	Symptomatic *n* = 66	Asymptomatic *n* = 30	OR	*P*_*c*_
Genotypes				
*HP 1-1 *	5 (3)	3 (1)	1.39	ns
*HP 2-1 *	24 (16)	80 (24)	0.08	ns
*HP 2-2 *	71 (47)	17 (5)	12.37	0,000003∗
Alleles				
*HP1 *	17 (22)	43 (26)	0.26	0,00016∗
*HP2 *	83 (110)	57 (34)	3.82	0,00016∗

*Note*. The genotype and allelic frequencies are expressed in percentages, followed by the number of individuals or chromosomes in parentheses. OR: odds ratio, *P*_*c*_: corrected *P* values, ns: not significant, ∗significant.
